# Preclinical Investigation of Alpinetin in the Treatment of Cancer-Induced Cachexia *via* Activating PPARγ

**DOI:** 10.3389/fphar.2021.687491

**Published:** 2021-05-21

**Authors:** Yujie Zhang, Yuxin Zhang, Yichen Li, Li Zhang, Shiying Yu

**Affiliations:** ^1^ Department of Oncology, Tongji Medical College, Tongji Hospital, Huazhong University of Science and Technology, Wuhan, China; ^2^ Hepatic Surgery Center, Tongji Medical College, Tongji Hospital, Huazhong University of Science and Technology, Wuhan, China

**Keywords:** alpinetin, cancer cachexia, skeletal muscle, C2C12 myotube, PPARγ, GW9662

## Abstract

The ongoing loss of skeletal muscle is a central event of cancer cachexia, and its consequences include adverse effects on patient’s quality of life and survival. Alpinetin (Alp), a natural plant-derived flavonoid obtained from *Alpinia katsumadai* Hayata, has been reported to possess potent anti-inflammatory and antitumor activities. This study aimed to explore the therapeutic effect and underlying mechanism of Alp in the prevention of cancer cachexia. We found that Alp (25–100 μM) dose-dependently attenuated Lewis lung carcinoma–conditioned medium-induced C2C12 myotube atrophy and reduced expression of the E3 ligases Atrogin-1 and MuRF1. Moreover, Alp administration markedly improved vital features of cancer cachexia *in vivo* with visible reduction of the loss of tumor-free body weight and wasting of multiple tissues, including skeletal muscle, epididymal fat, and decreased expression of Atrogin-1 and MuRF1 in cachectic muscle. Alp suppressed the elevated spleen weight and serum concentrations of tumor necrosis factor-α (TNF-α), interleukin (IL)-1β, and IL-6. Further, Alp treatment remained protective against cancer cachexia in the advanced stage of tumor growth. Molecular docking results suggested that Alp was docked into the active site of PPARγ with the docking score of –7.6 kcal/mol, forming a hydrogen bond interaction with PPARγ protein amino acid residue HIS449 with a bond length of 3.3 Å. Mechanism analysis revealed that Alp activated PPARγ, resulting in the downregulated phosphorylation of NF-κB and STAT3 *in vitro* and *in vivo*. PPARγ inhibition induced by GW9662 notably attenuated the improvement of Alp on the above cachexia phenomenon, indicating that PPARγ activation mediated the therapeutic effect of Alp. These findings suggested that Alp might be a potential therapeutic candidate against cancer cachexia.

**GRAPHICAL ABSTRACT:**
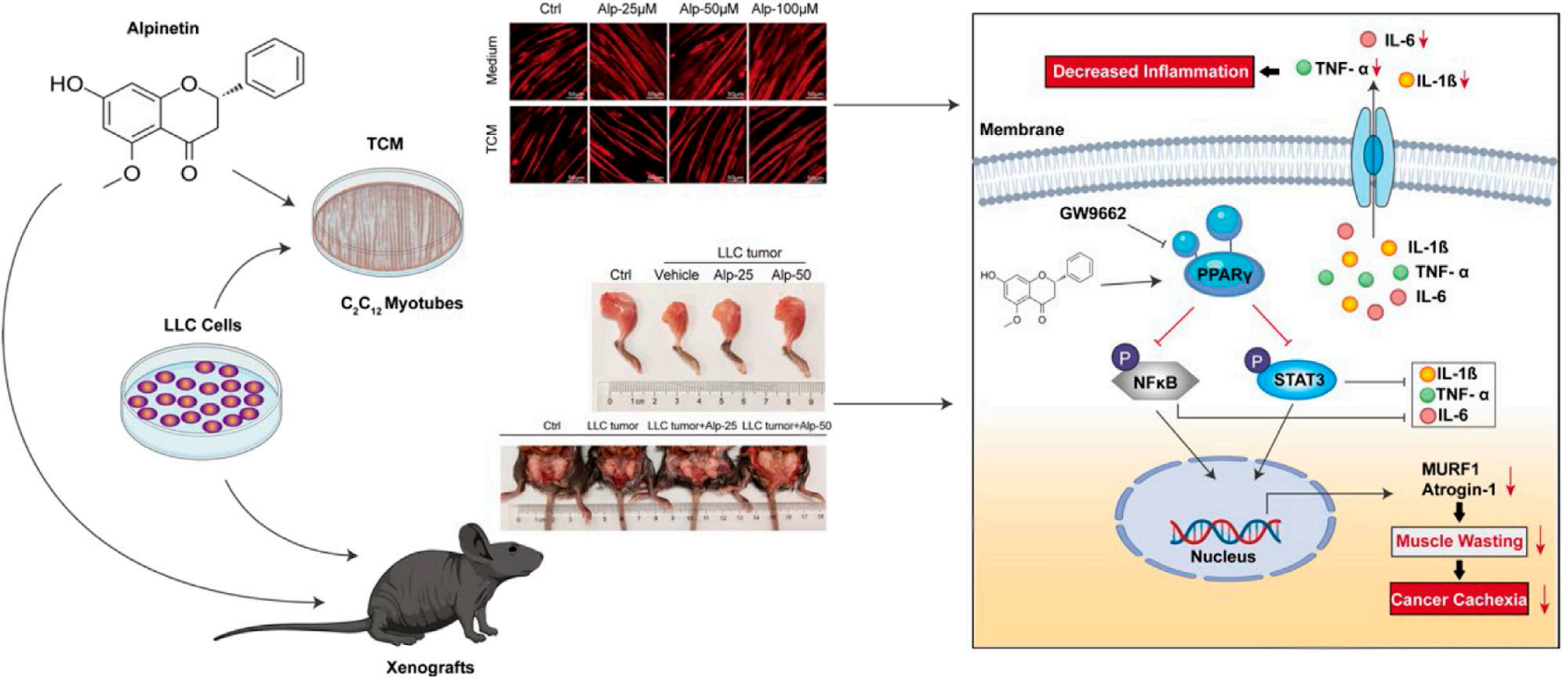


## Introduction

Cancer cachexia is a multifactorial syndrome marked by an ongoing depletion of skeletal muscle mass (with or without loss of fat mass) that cannot be entirely reversed by conventional nutritional support and leads to progressive functional impairment (Biswas and Acharyya, 2020). Loss of skeletal muscle is a key feature of the condition, and its consequences include significant adverse effects on the response to anticancer treatment, quality of life, and survival of patients (Fearon et al., 2013; Baracos et al., 2018; Boehm et al., 2020). Attenuating muscle atrophy is an important part to treat and alleviate cancer cachexia (Dutt et al., 2015; Ebner et al., 2020). However, no approved drug for the treatment of cancer-related muscle atrophy is available (Baracos et al., 2019), so it is necessary to find effective management.

Skeletal muscle atrophy is mainly mediated by pro-inflammatory cytokines and tumor-related mediators, which can activate the catabolic pathway of skeletal muscle. There are many signaling pathways involved, such as nuclear factor-kappa B (NF-κB) (Kuroda et al., 2005; Op Den Kamp et al., 2013), signal transducer and activator of transcription 3 (STAT3) (Zhang et al., 2013; Zimmers et al., 2016; Mulder et al., 2020; Shukla et al., 2020), transforming growth factor-beta (TGF-β) (Lima et al., 2019), and mitogen-activated protein kinase/extracellular signal–regulated kinase (MEK/ERK) signaling pathways (Quan-Jun et al., 2017). Tumor or immune cells often release some substances during tumor growth process, including tumor necrosis factor-α (TNF-α), interleukin-1β (IL-1β), IL-6, and interferon-gamma (IFN-γ) (Johnston et al., 2015), to activate the above signaling pathways in muscle cells, and then promote the occurrence and development of cachexia. TNF-α can bind to type I TNFR on the surface of muscle cells, activating the NF-κB signaling pathway(Zhao et al., 2015; Patel and Patel, 2017), and IL-6 can activate the STAT3 signaling pathway (Bonetto et al., 2012; Zimmers et al., 2016; Miller et al., 2017; Hu et al., 2019) to trigger muscle catabolism. Interferon-γ can synergistically promote the decomposition of muscle protein with TNF-α (Porporato, 2016). These factors lead to upregulation of E3 ubiquitin-protein ligases Trim63 (also known as MuRF1) and F-box only protein 32 (Fbxo32, also known as Atrogin-1), which are responsible for ubiquitination and proteasomal degradation of muscle protein (Argilés et al., 2014). The intervention of these signaling pathways or factors in animal or cell experiments can alleviate muscle wasting to a certain extent.

Peroxisome proliferator–activated receptors are nuclear receptors that regulate some cellular and metabolic processes, and consist of three types, namely, PPARα, PPARδ (or β), and PPARγ (Aljada et al., 2008). Lately, there has been growing attention to exploring the ability of PPARγ in inflammation, lipid metabolism, and tumor growth (Sawayama et al., 2014), especially in cancer cachexia (Beluzi et al., 2015; Jiang et al., 2015). Activation of PPARγ exerts an anti-inflammatory effect by suppressing the NF-κB signaling pathway and inhibiting IL-6 and other inflammatory factor production by regulating the STAT3 pathway (Yu et al., 2008; Gendy et al., 2021). Several studies have found that the mRNA and protein levels of PPARγ decreased significantly in visceral adipose tissue during the process of cancer cachexia (BING et al., 2006; Batista et al., 2012), demonstrating that the disorder of the PPARγ signaling pathway in lipid metabolism aggravated the development of cachexia. In addition, recent data showed that the expression level of PPARγ in skeletal muscles of tumor-bearing mice was markedly decreased, suggesting that it involved in the process of muscle atrophy (Moore-Carrasco et al., 2008). Therefore, we proposed that PPARγ may be a promising therapeutic target in the prevention and treatment of cancer cachexia.

**FIGURE 1:**
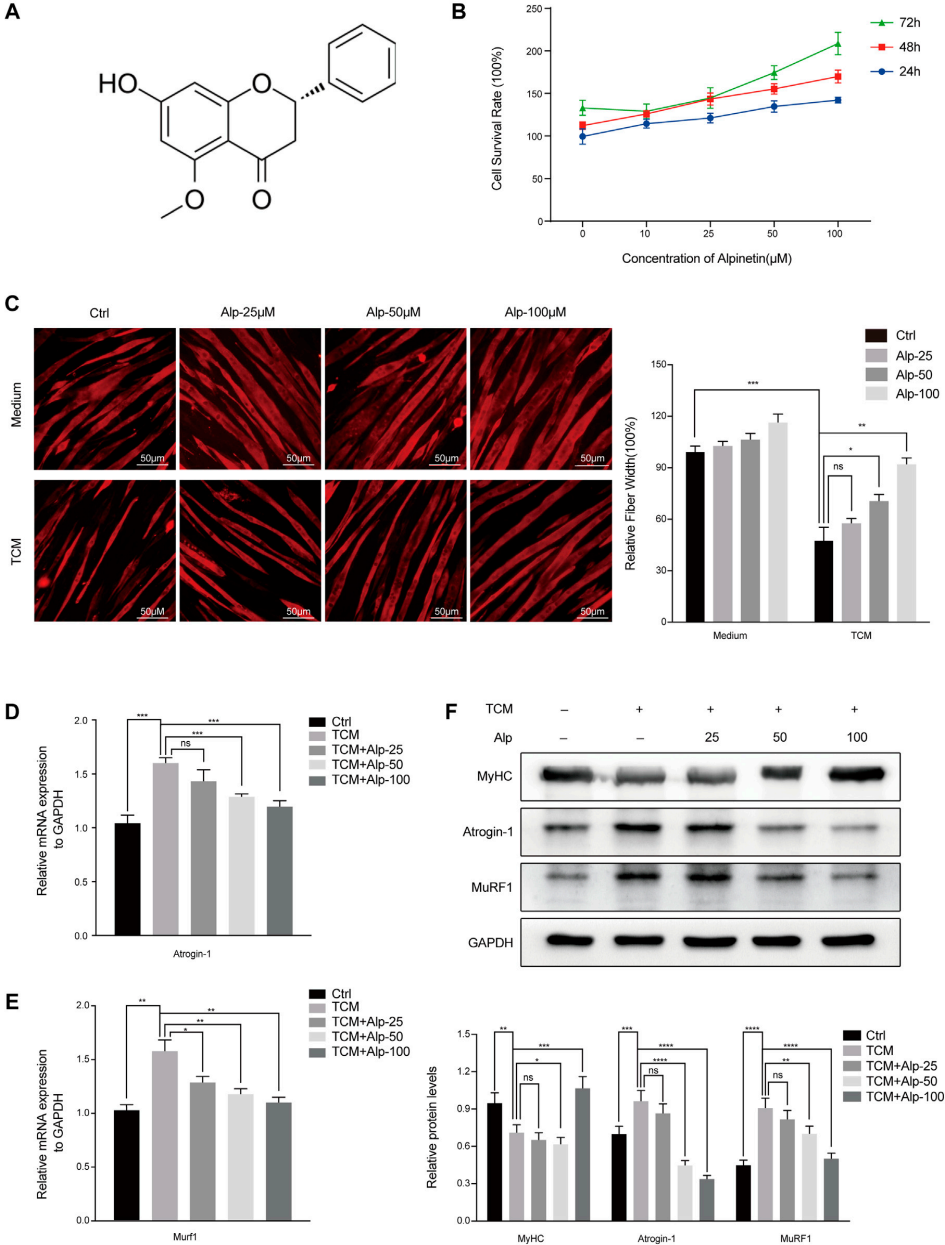
Alp reduces TCM-induced myotube atrophy. **(A)** The chemical structure of Alp. **(B)** The effect of Alp on C2C12 myotube viability. Myotubes were cultured with increasing concentrations of Alp (0, 10, 25, 50, and 100 μM) for 24, 48, and 72 h in the presence of Alp, and their viability was detected by CCK-8 assay. C2C12 myotubes were treated with Alp at various concentrations and stimulated by LLC TCM for 48 h. **(C)** Representative images of immunofluorescence staining for MyHC (red) are presented **(left)**. The relative fiber widths of each experiment were quantified **(right)**. mRNA levels of Atrogin-1 **(D)** and Murf1 **(E)** were analyzed by real-time PCR and normalized to GAPDH. **(F)** Western blot was used to detect the expression levels of indicated proteins, and the band densities were quantified and normalized with GAPDH. *p < 0.05, **p < 0.01, ***p < 0.001, ****p < 0.0001 vs. control. ns: not significant. *n* = 3.

Alpinetin (7-hydroxy-5-methoxyflavanone; molecular formula 1C6H14O4; Figure 1A) is a natural plant-derived flavonoid primarily obtained from *Alpinia katsumadai* Hayata (Lv et al., 2018) and has been reported to possess anti-inflammatory (Hu et al., 2013; He et al., 2016; Lv et al., 2018) and antitumor (Du et al., 2012; Wang et al., 2013; Wu et al., 2015; Zhao et al., 2018) activities, partly the roles *via* activating PPARγ. However, the effect of Alp on cancer cachexia and its underlying molecular mechanism is still unclear at present, so it was explored in this work. We found that Alp could reverse Lewis lung carcinoma tumor cell–conditioned medium-induced myotube atrophy and inhibit MuRF1 and Atrogin-1 expression *in vitro*. Alp can also alleviate the animal cachexia caused by LLC tumor, prevent muscle wasting, reduce the expression of MuRF1 and Atrogin-1 *in vivo*, and decrease the level of cytokines in serum. More importantly, Alp treatment maintained protective against cachexia in the late stage of tumor growth. Molecular docking analysis revealed that Alp bound with the active site of PPARγ, forming a hydrogen bond interaction with PPARγ protein amino acid residue HIS449. Mechanistically, Alp activated PPARγ, thereby attenuating NF-κB (p65 subunit) and STAT3 phosphorylation in myotube and skeletal muscle. The above phenomenon triggered by Alp could be recovered by PPARγ inhibitor (GW9662), suggesting that PPARγ activation mediated the therapeutic role of Alp. Together, these findings provide new insights into the anti-cachexia effect of Alp, which may be a promising candidate against cancer cachexia.

## Materials and Methods

### Reagents and Antibodies

The antibodies targeting p-STAT3 (Tyr705) (#9145), STAT3 (#9139), p-NF-κB p65 (Ser536) (#3033), and NF-κB p65 (#8242) were obtained from Cell Signaling Technology (Beverly, MA); the antibody against GAPDH (#60004-1-Ig) was obtained from Proteintech (Wuhan, China); the antibodies against MuRF1 (ab172479), Atrogin-1 (ab168372), and PPARγ (ab59256) were purchased from Abcam (Cambridge, MA); the antibody against myosin heavy chain (MyHC, MAB4470) was obtained from R&D Systems (Minneapolis, MN); Alp and GW9662 were obtained from MedChemExpress (NJ, United States).

### Cell Cultures and the Conditioned Medium Preparation

Murine C2C12 myoblast and LLC cells (Cell Bank of Chinese Academy of Sciences) were cultured in the DMEM (Hyclone, United States) supplemented with 10% fetal bovine serum (FBS, Gibco, United States) and 1% penicillin/streptomycin (Hyclone, United States). To differentiate into myotube, C2C12 myoblasts were proliferated to 80–90% confluence and replaced with DMEM supplemented with 2% horse serum (Gibco, United States) as a differentiation medium (DM). The DM was renewed every day, and myotubes were completely differentiated on days 5–6.

LLC cells were seeded in 10 mm dishes and grown to 80–90% confluence. The medium was changed to FBS-free DMEM for 48 h, and cell supernatant was collected and centrifuged at 3,000 rpm for 20 min at 4°C. The supernatant was filtered through a 0.22-μm sterile filter. The filtered medium was called LLC-conditioned medium (CM) and should be used immediately or stored at – 80°C. The LLC tumor–conditioned medium (TCM) consisted of CM and DMEM diluted at 1:1 combined with 2% horse serum, which was the factor inducing C2C12 myotube atrophy.

### Cell Viability Assay

C2C12 myoblasts (1 × 104 cells/well) were plated into 96-well plates and differentiated into myotube. The myotubes were treated with various concentrations of Alp on triplicate wells for 24, 48, or 72 h. Cell viability was detected using Cell Counting Kit-8 assay (CCK-8, MedChemExpress, United States) by adding 10 μL CCK-8 reagent to each well for 2 h at 37°C, and the absorbance was measured at 450 nm by the microplate reader (BioTek, United States).

### Immunofluorescence

C2C12 myotubes were fixed in 4% paraformaldehyde, permeabilized with 0.1% Triton X‐100 (Servicebio, Wuhan, China), blocked with 0.5% BSA (Sigma, United States), and incubated with MyHC antibody and secondary antibody (Proteintech, Wuhan, China), followed by staining with 4′, 6‐diamidino‐2‐phenylindole (DAPI, Boster, Wuhan, China). Following this, myotubes were visualized by a fluorescence microscope (Leica, Germany), and images were quantitated using ImageJ software.

### Animal Experimentation

Investigation has been conducted in accordance with the ethical standards, and according to the Declaration of Helsinki and national and international guidelines. All animal care and procedures were conducted according to the ARRIVE (Animal Research: Reporting of *In Vivo* Experiments) guidelines and the AVMA (the American Veterinary Medical Association) euthanasia guidance and were approved by the Animal Ethics Committee of Tongji Hospital, Tongji Medical College, the Huazhong University of Science and Technology.

**FIGURE 2:**
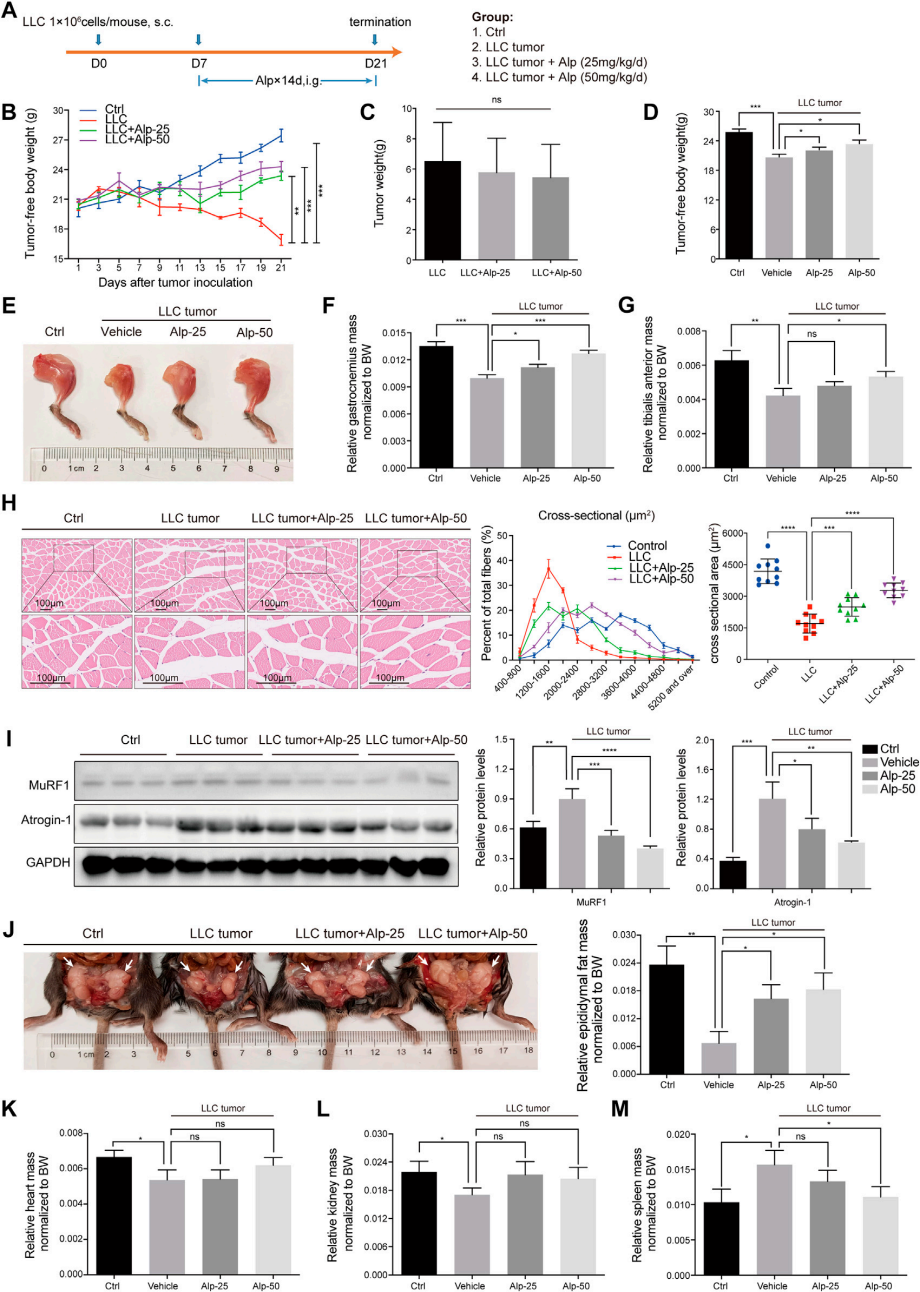
Alp prevents weight loss and attenuates muscle wasting in LLC tumor–bearing mice. **(A)** Schematic presentation of the animal experiment design. The effects of Alp on the main parameters of cachexia were observed, including **(B–D)** tumor-free body weight, **(C)** tumor weight, **(E–G)** gastrocnemius and tibialis anterior muscles mass, **(J)** epididymal fat mass, and **(K–M)** heart, kidney, and spleen mass (BW: body weight). **(H)** Gastrocnemius muscle was examined histologically by HE staining and cross-sectional area analysis. **(I)** Protein levels of Atrogin-1 and MuRF1 in gastrocnemius muscles were evaluated by Western blot, and the densities were calculated and normalized with GAPDH. *p < 0.05, **p < 0.01, ***p < 0.001, ****p < 0.0001 vs. control. ns: not significant. *n* = 10 mice/group.

Five‐week‐old male C57BL/6 mice were inoculated with LLC cells (1 × 106 cells/100 μL PBS) subcutaneously into the right flanks. For all animal experiments, the study endpoint was day 21 after LLC cell inoculation. In the first study, we assessed the therapeutic effects of Alp on tumor-induced cachexia. Alp was orally administrated starting on day 7 after tumor inoculation. The experiment protocol was shown in Figure 2A. The mice were randomized into four groups with ten animals in each group: normal control group; LLC tumor–bearing/vehicle group; LLC tumor–bearing and 25 mg/kg Alp-treated group; and LLC tumor–bearing and 50 mg/kg Alp-treated group. The normal control and LLC tumor–bearing/vehicle group received an equal volume of corn oil. The dose selection of Alp was based on the xenograft tumor model (50 mg/kg/d) (Wu et al., 2015) and inflammatory disease (25, 50, and 100 mg/kg/d) (He et al., 2016). The body weights and tumor sizes were measured every two days. Tumor weights (mg) were computed using the formula: 0.52 × tumor length × tumor width^2^ (Chen et al., 2020). The body weight was calculated by subtracting the tumor weight from the total animal weight. After 14 days of treatment, the weights of tumors, muscles, and tissues (epididymal fat, kidney, spleen, and heart) were then determined. The blood was collected into tubes, and the serum was prepared within 1 h for measurement of pro-inflammatory cytokines. Some muscles were quickly frozen in liquid nitrogen, and the remaining were fixed in 4% polyformaldehyde for subsequent analysis.

The second study investigated Alp-related mechanisms against cancer cachexia. Fifty male mice were randomly divided into five groups: normal control and corn oil–treated group; LLC tumor–bearing and corn oil–treated group; LLC tumor–bearing and 50 mg/kg Alp (i.g.)-treated group; LLC tumor–bearing and 1 mg/kg GW9662 (i.p.)-treated group; LLC tumor–bearing and 50 mg/kg Alp; and 1 mg/kg GW9662-treated group. The dose selection of GW9662 was based on the systemic inflammatory response in cancer cachexia (1 mg/kg/d, i. p.) (JIANG et al., 2016). The observed data and collected tissues were the same as those in the first study.

The third animal study explored the efficacy of delayed treatment with 50 mg/kg Alp (i.g.) during cachexia process. Alp administration was started at 6, 10, or 14 days after LLC cell injection. The main features and samples were obtained by following the same methods as in the first study.

### Histological Examination

Three gastrocnemius muscles were selected randomly from each group, fixed with 4% paraformaldehyde, and embedded in paraffin. The transverse serial sections of gastrocnemius muscles were stained with hematoxylin and eosin (H and E). Images of muscle sections were captured by the Leica microscope. The cross-sectional areas of the fibers were analyzed by ImageJ software (NIH, United States), and Image-Pro Plus 6.0 (Media Cybernetics, United States) was used to calculate fiber types. The cross-sectional areas of gastrocnemius muscles were quantified from approximately 50 fibers per mouse/muscle in each group.

### Western Blot Analysis

C2C12 myotube and gastrocnemius muscle samples were lysed by RIPA buffer (Beyotime, Shanghai, China), including PMSF and Phosphatase Inhibitor Cocktail (MedChemExpress, United States) for 30 min on ice. After centrifugation at 4°C, 12,000 g for 20 min, the protein was collected, and its concentration was measured by BCA Protein Assay Kits (Beyotime, Shanghai, China). Equal amounts of proteins were separated by 10% SDS‐PAGE gels and transferred onto PVDF membranes (Millipore, Billerica, United States). The membranes were blocked in 5% BSA for 1 h at room temperature, incubated with primary antibodies overnight at 4°C, and followed by incubation with corresponding secondary antibodies (Boster, Wuhan, China) at room temperature for 1 h. Finally, the immunoblots were visualized by ECL kits (Thermo Fisher Scientific, United States). Images were captured using Syngene G (AlphaMetrix Biotech, Hesse, Germany), and the density of each blot band was quantified by ImageJ software.

### RNA Extraction and Quantification of mRNA Expression

Total RNA was extracted from C2C12 myotube using RNAiso Plus (Takara, Japan), and cDNA was synthesized by 1 μg RNA using PrimeScript RT Master Mix (RR036A; Takara, Japan) as described in the manufacturer's instructions. The mRNA expression levels of Murf1, Atrogin-1, and PPARγ were detected with a StepOne™ Real‐Time PCR System (ABI, CA, United States). Values were normalized to the reference GAPDH gene, and the relative expressions were analyzed by the ΔΔCt method. The primer sequences are the following: Murf1 (forward: CCA​GGC​TGC​GAA​TCC​CTA​C; reverse: ATT​TTC​TCG​TCT​TCG​TGT​TCC​TT), Atrogin-1 (forward: CAG​CTT​CGT​GAG​CGA​CCT​C; reverse: GGC​AGT​CGA​GAA​GTC​CAG​TC), PPARγ (forward: GGA​AGA​CCA​CTC​GCA​TTC​CTT; reverse: GTA​ATC​AGC​AAC​CAT​TGG​GTC​A), and GAPDH (forward: AGG​TCG​GTG​TGA​ACG​GAT​TTG; reverse: GGG​GTC​GTT​GAT​GGC​AAC​A).

### ELISA Assay

The animal blood sample was centrifuged at 3,000 rpm for 20 min to separate serum. According to the manufacturer's protocol, the levels of TNF-α, IL-1β, and IL-6 in serum were detected by the commercially available mouse ELISA kits purchased from Neobioscience (Shenzhen, China), and the serum of each animal was determined in duplicate.

### Molecular Docking

In order to elucidate the binding mode between PPARγ protein and Alp at the molecular level, molecular docking was performed in the AutoDock Vina 1.1.2. The exhaustiveness was set as 20, and the other options were set as default settings. The crystal structure of PPARγ protein was downloaded from the RCSB Protein Data Bank (www.rcsb.org), and the structure of Alp was downloaded from the PubChem database (https://pubchem.ncbi.nlm.nih.gov/). Prior to docking, the molecular structures were processed by AutoDockTools 1.5.6.

### Statistical Analysis

All *in vitro* experiments were repeated three times. Data and results were presented as mean ± SD, and Student’s t-test or one-way ANOVA followed by the Dunnett *post hoc* test was used to assess the statistical significance. Differences were defined as statistically significant at *p* < 0.05.

## Results

### Alpinetin Reduces TCM-Induced Myotube Atrophy

Muscle protein wasting is a critical problem of cancer cachexia. To clarify the anti-cachexia role of Alp, we initially explored its impact on C2C12 myotube atrophy induced by LLC TCM. There was no toxicity of Alp at concentrations below 100 μM for 24, 48, and 72 h to C2C12 myotube using CCK8 assay (Figure 1B), so 25, 50, and 100 μM were chosen for the following experiments. Results showed that Alp (25–100 μM) dose-dependently attenuated TCM-induced myotube atrophy, as evaluated by morphology and the width of myotube (Figure 1C). Moreover, Alp treatment drastically inhibited TCM-induced expression of Atrogin-1 and Murf1 mRNA (Figures 1D,E) and protein (Figure 1F), as well as increased the protein expression of MyHC in C2C12 myotube (Figure 1F). These results demonstrated that Alp protected the myotube atrophy induced by TCM and reduced ubiquitin-mediated muscle protein degradation.

### Alp Inhibits Cancer Cachexia and Attenuates Muscle Wasting in LLC Tumor–Bearing Mice

We then evaluated the therapeutic effect of Alp in the animal model of cancer cachexia using LLC Lewis lung carcinoma (Figure 2A). Compared with the control group, LLC tumor–bearing mice showed marked loss of tumor-free body weight (Figures 2B,D) and weight of gastrocnemius, tibialis anterior muscle, and epididymal fat, as well as heart and kidney weight. In contrast, the weight of the spleen was significantly increased (Figures 2E–G,J–M). Alp distinctly improved the main characteristics of cancer cachexia in a dose-dependent manner, considerably increasing the tumor-free body weight and weights of gastrocnemius, tibialis anterior muscle, and epididymal fat mass compared to the LLC tumor group (Figure 2B,D-G,J). Moreover, Alp can reduce the weight of the spleen, and this effect was statistically significant at the dose of 50 mg/kg, which may be related to the inhibition of the inflammatory response by Alp treatment (Figure 2M). The raw values of muscle, epididymal fat, and organ weights are presented in Supplementary Figure S1. HE staining and cross-sectional area analysis of gastrocnemius muscle demonstrated myofibers were wasted in the LLC tumor group, while it was reversed by Alp treatment in a dose-dependent manner (Figure 2H). As confirmed by the Western blot, Alp treatment induced an apparent reduction of MuRF1 and Atrogin-1 expression in gastrocnemius muscle in a dose-dependent manner (Figure 2I). Unfortunately, LLC tumor growth was not affected by Alp at doses of 25 and 50 mg/kg (Figure 2C). These results indicated that the effect of Alp in preventing cancer cachexia and attenuating muscle atrophy in LLC tumor–bearing mice was not the result of tumor burden reduction.

### Alp Increases PPARγ Expression, Inhibits Phosphorylation of STAT3 and NF-κB, and Reverses Elevated Serum Cytokines in the Cancer Cachectic Model

**FIGURE 3:**
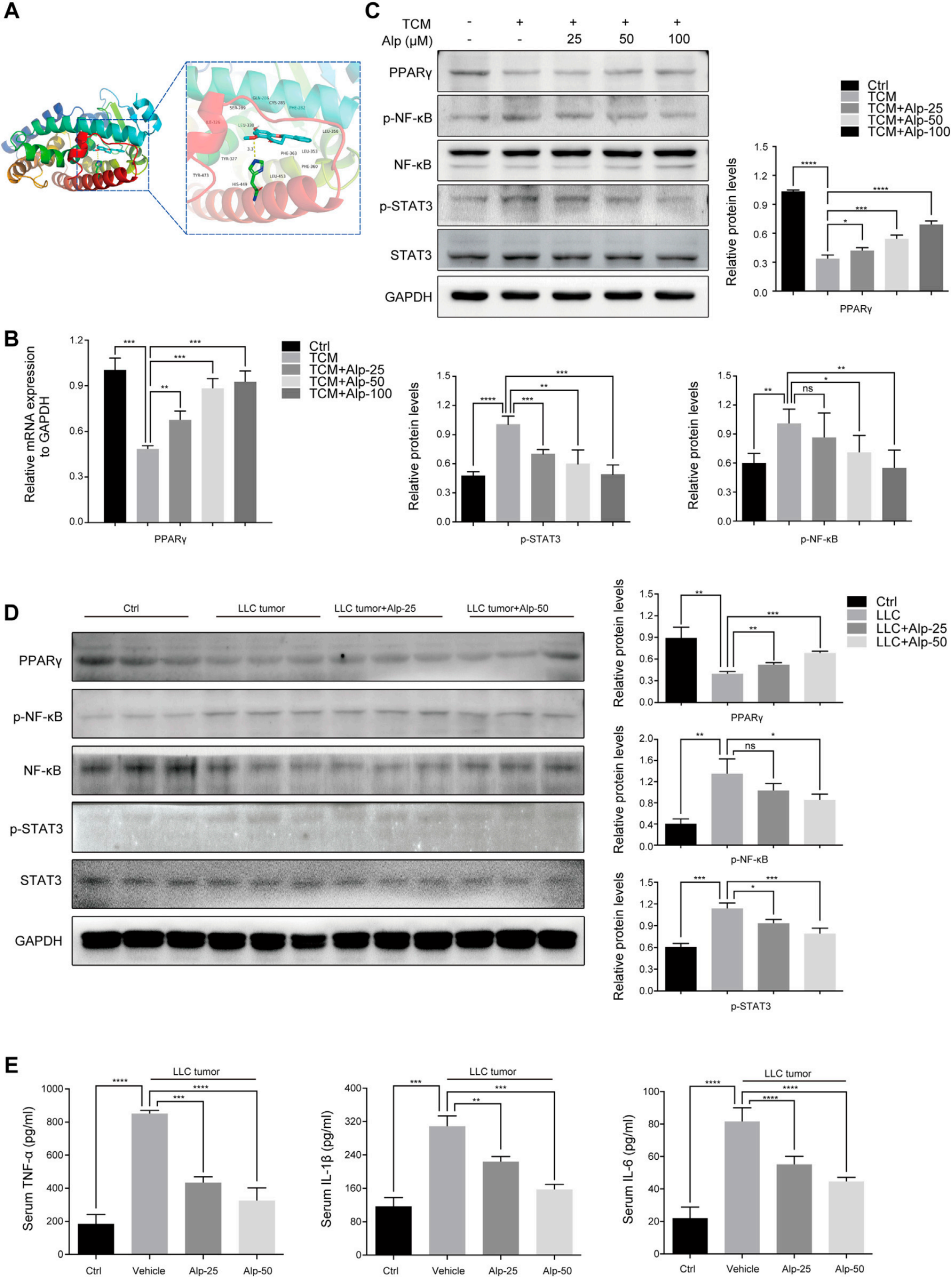
Alp increases PPARγ expression, inhibits phosphorylation of STAT3 and NF-κB, and reverses elevated serum cytokines in the cancer cachectic model. **(A)** Molecular docking analysis of the binding mode between Alp and the active site of PPARγ protein. **(B)** In the C2C12 myotube model, the mRNA expression of PPARγ was analyzed by real-time PCR, and **(C)** the indicated proteins were detected by Western blot. The band densities were quantified and normalized with GAPDH or the non-phosphorylated protein forms. **(D)** In the gastrocnemius muscles of the animal model, the expression levels of indicated proteins were examined by Western blot, and the densities were quantified and normalized with GAPDH or the non-phosphorylated protein forms. **(E)** Pro-inflammatory cytokines of TNF-α, IL-1β, and IL-6 were analyzed by ELISA in the serum of the cachexia mice model. *p < 0.05, **p < 0.01, ***p < 0.001, ****p < 0.0001 vs. control. ns: not significant. *n* = 3.

To investigate the possible mechanisms underlying the anti-cachexia role of Alp, we explored the interaction mode between PPARγ protein and Alp at the molecular level, and the effects of PPARγ activation and alterations of signaling pathways related to muscle wasting. The molecular docking results indicated that Alp was docked into the active site of PPARγ with the docking score of – 7.6 kcal/mol. The theoretical binding mode was shown in Figure 3A. Alp and PPARγ protein amino acid residue HIS449 form a hydrogen bond with a bond length of 3.3 Å, which makes the protein and Alp form a stable complex. The docking results lay a theoretical foundation for the further study of Alp in cancer cachexia. In the C2C12 myotube atrophy model, Alp upregulated the mRNA and protein levels of PPAR-γ and suppressed the protein expression of phosphorylation of STAT3 and NF-κB in a dose-dependent manner (Figures 3B,C). Consistent with the cellular data, Alp treatment also restored the decreased PPAR-γ expression levels and inhibited STAT3 and NF-κB phosphorylation in gastrocnemius muscles of LLC tumor–bearing mice as shown by the Western blot (Figure 3D). Furthermore, it was found that the LLC tumor group showed high levels of TNF-α, IL-1β, and IL-6 (Figure 3E), and Alp reduced their serum concentrations in a dose-dependent manner.

### Alp Improves Muscle Wasting Through PPARγ Activation

**FIGURE 4:**
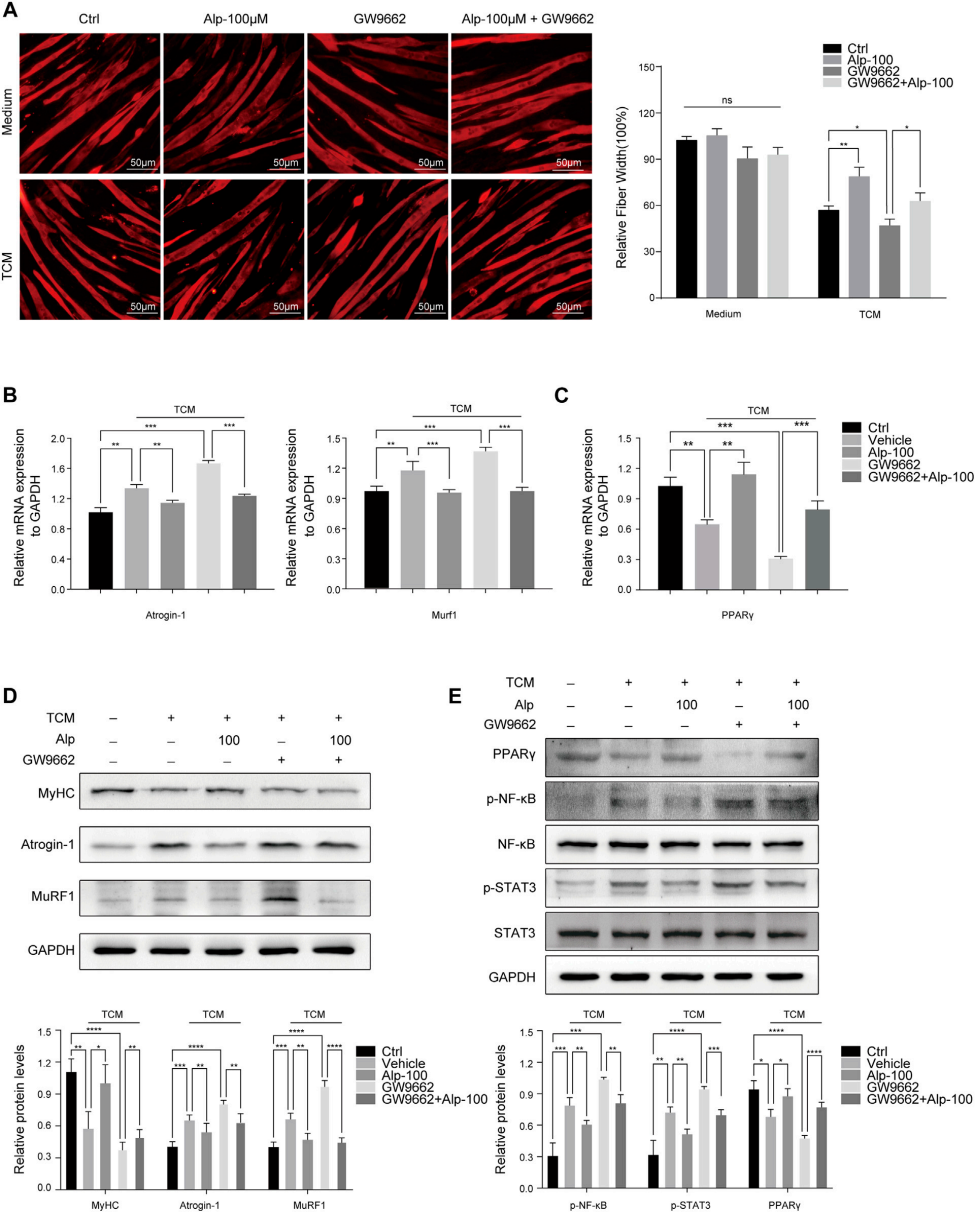
Alp improves TCM-induced myotube wasting through PPARγ activation. C2C12 myotubes were pretreated with GW9662 (10 μM) for 30 min and then stimulated by Alp (100 μM) and LLC TCM for 48 h. **(A)** Representative immunofluorescence images for MyHC (red) are displayed **(left)**, and the fiber widths were measured **(right)**. **(B,C)** The mRNA expressions of Atrogin-1, Murf1, and PPARγ were detected by real-time PCR and normalized with GAPDH. **(D,E)** The levels of indicated proteins in myotube were detected by Western blot, and the band densities were measured and normalized with GAPDH or the non-phosphorylated protein forms. *p < 0.05, **p < 0.01, ***p < 0.001, ****p < 0.0001 vs. control. ns: not significant. *n* = 3.

To explore whether PPARγ activation mediates the therapeutic effect of Alp on cancer cachexia, we used the PPARγ inhibitor GW9662 (10 μM, the dose selection was based on the tumor cell model (Seargent et al., 2004)) and assessed its impact on myotube atrophy *in vitro*. Compared with the control group, the inhibition of PPARγ resulted in a marked decrease in fiber width and significant myotube atrophy. Alp treatment can reverse myotube atrophy caused by TCM, while the improvement effect was significantly weakened by PPARγ inhibition (Figure 4A). GW9662 attenuated the inhibition of Alp on the expression of Atrogin-1 and MuRF1, as well as the protein level of MyHC in C2C12 myotube (Figure 4B,D). Besides, GW9662 also reduced the upregulation of PPAR *γ* mRNA and protein expression and the inhibition of STAT3 and NF-κB phosphorylation by Alp in the myotube atrophy model (Figure 4 C,E).

**FIGURE 5:**
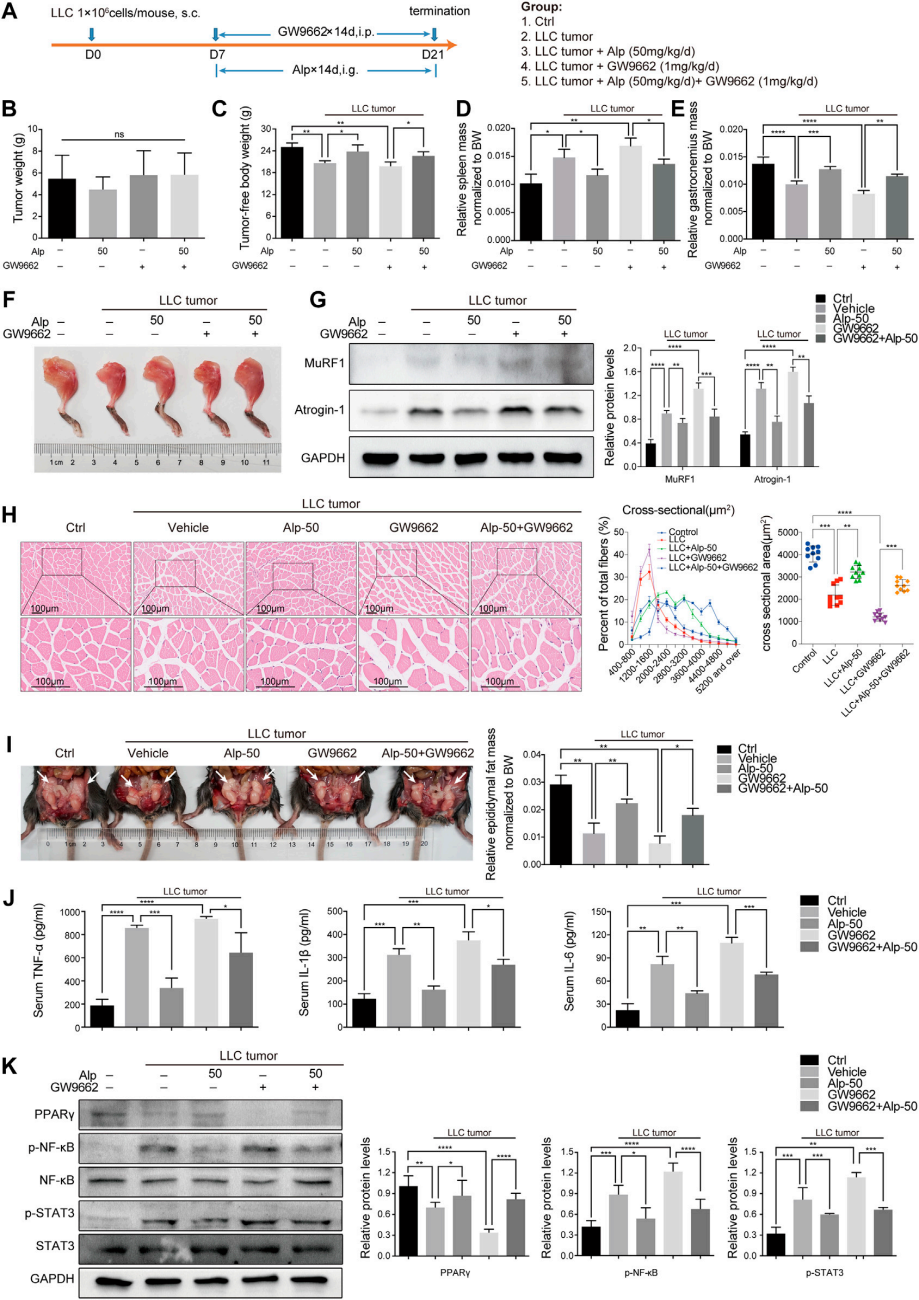
Alp activates PPARγ in muscles of LLC tumor–bearing mice. **(A)** Schematic presentation of the animal experiment protocol. Effects of PPARγ on **(B)** tumor weight, **(C)** tumor-free body weight, **(D)** spleen mass, **(E,F)** gastrocnemius muscle mass, and **(I)** epididymal fat mass of LLC tumor–bearing cachexia mice were analyzed (BW: body weight). **(G–K)** The expressions of target proteins in gastrocnemius muscle were detected by Western blot, and the densities were calculated and normalized with GAPDH or the non-phosphorylated protein forms. **(H)** Gastrocnemius muscle atrophy was showed by HE staining and cross-sectional area analysis. **(J)** Pro-inflammatory cytokines of TNF-α, IL-1β, and IL-6 were analyzed by ELISA in the serum of the animal model. *p < 0.05, **p < 0.01, ***p < 0.001, ****p < 0.0001 vs. control. ns: not significant. *n* = 10 mice/group.

To further confirm the role of PPARγ, we used GW9662 and evaluated its function *in vivo*. The schematic diagram of the experimental design was shown (Figure 5A). GW9662 reduced the increase in tumor-free body weight and decrease in spleen weight induced by Alp, and aggravated gastrocnemius and epididymal fat mass wasting in LLC tumor–bearing mice (Figures 5C–F,H,I). However, GW9662 had no significant effect on tumor weight (Figure 5B). Effects of PPARγ on tumor-free body weight during the 21-day study, the weight of the heart and kidney, and tibialis anterior muscle mass of LLC tumor–bearing cachexia mice were shown in Supplementary Figure S2. We found that GW9662 had no significant effect on the weight of the heart and kidney, while it decreased the weight of the tibial anterior muscle. The inhibitory effect of Alp on MuRF1 and Atrogin-1 expression in the gastrocnemius muscle was also weakened after GW9662 administration (Figure 5G). GW9662 treatment distinctly aggravated the decreased PPAR-γ expression levels and increased STAT3 and NF-κB phosphorylation in gastrocnemius muscles of LLC tumor–bearing mice as shown by the Western blot (Figure 5K). Furthermore, it was found that GW9662 attenuated the inhibition of Alp on the release of TNF-α, IL-1β, and IL-6 in serum (Figure 5J). In conclusion, these data suggested that PPARγ activation mediated the anti-cachexia effect of Alp.

### Effects of Delayed Treatment With Alp on Cancer Cachexia

Early treatment of cancer cachexia is not clinically practicable owing to the limitation of early diagnosis. In the preceding animal experiments, Alp was administered early in disease progression when apparent signs of wasting were undetectable. To explore whether later initiation of Alp treatment remains protective against cancer cachexia, LLC tumor–bearing mice were treated with Alp starting at 6, 10, and 14 days after tumor cell injection.

**FIGURE 6:**
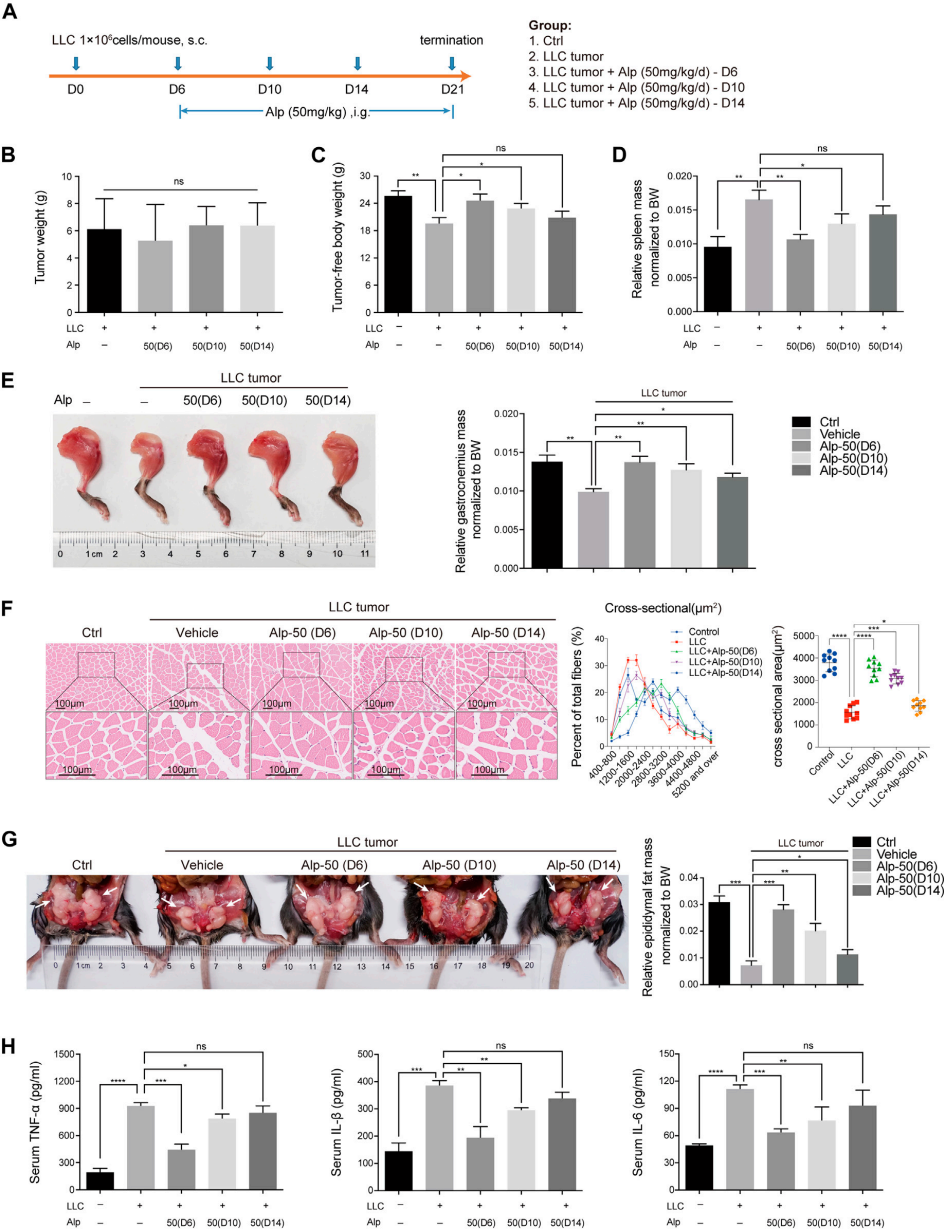
Effects of delayed administration with Alp until advanced stages of tumor and cachexia progression in LLC tumor–bearing mice. **(A)** Schematic presentation of the experimental design. The delayed treatment role of Alp in **(B)** tumor weight, **(C)** tumor-free body weight, **(D)** spleen mass, **(E)** gastrocnemius muscle mass, and **(G)** epididymal fat mass of cachexia mice were explored (BW: body weight). **(F)** Gastrocnemius muscle wasting was presented by HE staining and cross-sectional area analysis. **(H)** Pro-inflammatory cytokines of TNF-α, IL-1β, and IL-6 were analyzed by ELISA in the serum of cachexia mice. *p < 0.05, **p < 0.01, ***p < 0.001, ****p < 0.0001 vs. control. ns: not significant. *n* = 10 mice/group.

LLC tumor–bearing/vehicle mice lost 23.7% of tumor-free body weight by day 21, while treatment with Alp from day 6, 10, or 14 limited body weight loss to 4.2, 10.9, and 17.8%, respectively (Figure 6C), with no significant effect on tumor growth (Figure 6B). Alp preserved gastrocnemius wasting from day 6 or 10 (Figures 6E,F) and epididymal fat atrophy from day 6, 10, or 14 (Figure 6G). However, Alp had no significant effect on spleen weight from day 10 or 14 (Figure 6D). The delayed therapeutic effects of Alp on tumor-free body weight during the 21-day study, and the mass of the heart, kidney, and tibialis anterior muscle of cachexia mice were shown in Supplementary Figure S3. Results revealed that Alp had no crucial effect on the weight of the heart and kidney while increasing the tibial anterior muscle mass from day 6 or 10. Furthermore, it was found that Alp decreased the levels of TNF-α, IL-1β, and IL-6 in serum from day 6 or 10 (Figure 6H).

## Discussion

Cancer cachexia is a multifactorial wasting syndrome, and the ongoing loss of skeletal muscle has been recognized as a central event (Fearon et al., 2011; Fearon et al., 2013; Baracos et al., 2018). However, the nature of the crucial factors responsible for muscle wasting in cancer cachexia is still unclear, and the treatment methods are limited at present (Molfino et al., 2019).

Alp has been shown to have potential anti-inflammatory and antitumor activities (Wu et al., 2015; Lv et al., 2018), while its effect on cancer cachexia largely remains unclear. To investigate the role of Alp in cachexia, we preliminarily evaluated its impact on the C2C12 myotube atrophy model induced by LLC TCM. We found that Alp attenuated myotube atrophy in a dose-dependent manner as presented by morphological changes, muscle fiber size, and the reduced expression of Atrogin-1 and MuRF1. Moreover, Alp treatment dose-dependently improved the main features of cancer cachexia in LLC tumor–bearing mice, with increased tumor-free body weight and the mass of gastrocnemius muscle, tibialis anterior muscle, and epididymal fat. The myofiber size reduction of gastrocnemius muscle was restored, reflected by HE staining and the reduced level of Atrogin-1 and MuRF1. However, the anti-cachexic role of Alp seemed to be irrelevant to its antitumor effect because we found that Alp inhibited cancer cachexia with an insignificant effect on tumor weight. These data suggested that Alp could inhibit cancer cachexia progression and improve muscle wasting. More importantly, it is of great therapeutic significance that Alp administration at the late stage of tumor growth could delay muscle atrophy progression and prevent cachexia process in LLC tumor–bearing mice.

To evaluate the binding mode between PPARγ protein and Alp, molecular docking was performed. Results demonstrated that Alp binds to the active site of PPARγ with the docking score of – 7.6 kcal/mol and forms a hydrogen bond interaction with PPARγ protein amino acid residue HIS449 with a bond length of 3.3 Å. The detailed mechanism was elucidated that activating PPARγ by Alp treatment resulted in the downregulated phosphorylation of NF-κB (p65 subunit) and STAT3, two crucial regulatory pathways of cancer cachexia (Baracos et al., 2018). The C2C12 myotube depletion model and LLC tumor–bearing mice were used to observe the mediating effect of PPARγ. The findings revealed that myotube atrophy induced by LLC TCM was aggravated by PPARγ inhibitor, GW9662. Consistent with the cellular results, the anti-wasting effect of Alp in the animal model of cancer cachexia was also significantly reduced by GW9662. The inhibitory effects on Atrogin-1 and MuRF1 expression and phosphorylation of NF-κB (p65 subunit) and STAT3 were weakened after Alp treatment was combined with GW9662. Therefore, we conclude that Alp attenuates cancer cachexia and alleviates muscle wasting *via* activating PPARγ.

Cytokines produced by immune or tumor cells, including TNF-α, IL-1β, and IL-6, have been proven to cause muscle atrophy in cachexia patients (Baracos et al., 2018) and animal models (Tseng et al., 2015; Park et al., 2019). The results demonstrated that Alp dose-dependently decreased the levels of TNF-α, IL-1β, and IL-6 in the serum and reduced the spleen mass at the dose of 50 mg/kg in LLC tumor–bearing mice. These findings indicated that Alp has effects on immune or tumor cells, which may explain the phenomenon that the anti-wasting role of Alp was not wholly disappeared after it was combined with GW9662. However, how Alp regulates immune or tumor cells, and their interaction with muscle cells remain unknown.

In conclusion, these results suggest that Alp alleviates cancer cachexia progression and prevents muscle atrophy *via* activating PPARγ and thereby inhibiting phosphorylation of NF-κB and STAT3. Moreover, Alp treatment remains effective at the advanced stage of cancer wasting. These findings provide new insights for Alp in the treatment of cancer cachexia.

## Limitations

It is acknowledged that our study lacks the basis for the female animals, and the mice employed in this study were young (five weeks). Future studies should evaluate the Alp effects on female and sexually mature mice. Besides, Alp may have an effect on many related molecules and pathways in the cancer cachexia model. In this study, we focused on the role of PPARγ. As we have not explored other related molecules in this study, the percentage of the Alp effect attributed to PPARγ activation needs to be further explored. Finally, both the treatment time and the cachexia severity may have a certain impact on the results in the third animal experiment.

## Data Availability

The raw data supporting the conclusions of this article will be made available by the authors, without undue reservation.
