# Supplementary material for: Preclinical Investigation of Alpinetin in the Treatment of Cancer-Induced Cachexia via Activating PPARγ
Source: Front Pharmacol. 2021 May 21;12:687491. doi: 10.3389/fphar.2021.687491 (PMC8176100; doi:10.3389/fphar.2021.687491)
Supplement: Supplementary file 1 [file DataSheet1.docx]

| **Figure S1** Alp prevents weight loss and attenuates muscle wasting in LLC tumor-bearing mice. (A) Gastrocnemius and (B) tibialis anterior muscles mass, (C) epididymal fat mass, and (D-F) heart, kidney and spleen mass presented are raw values. n = 10 mice/group. 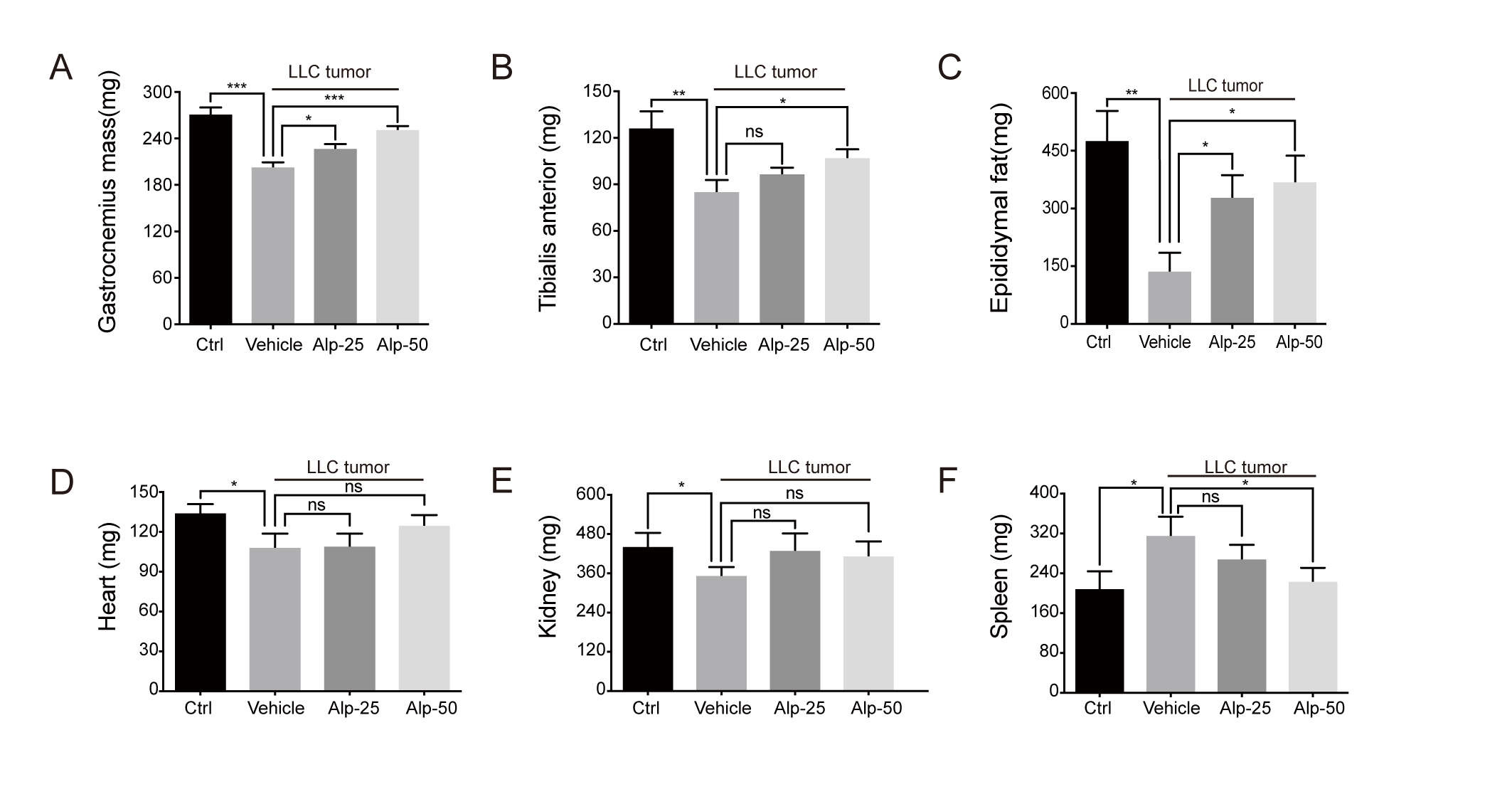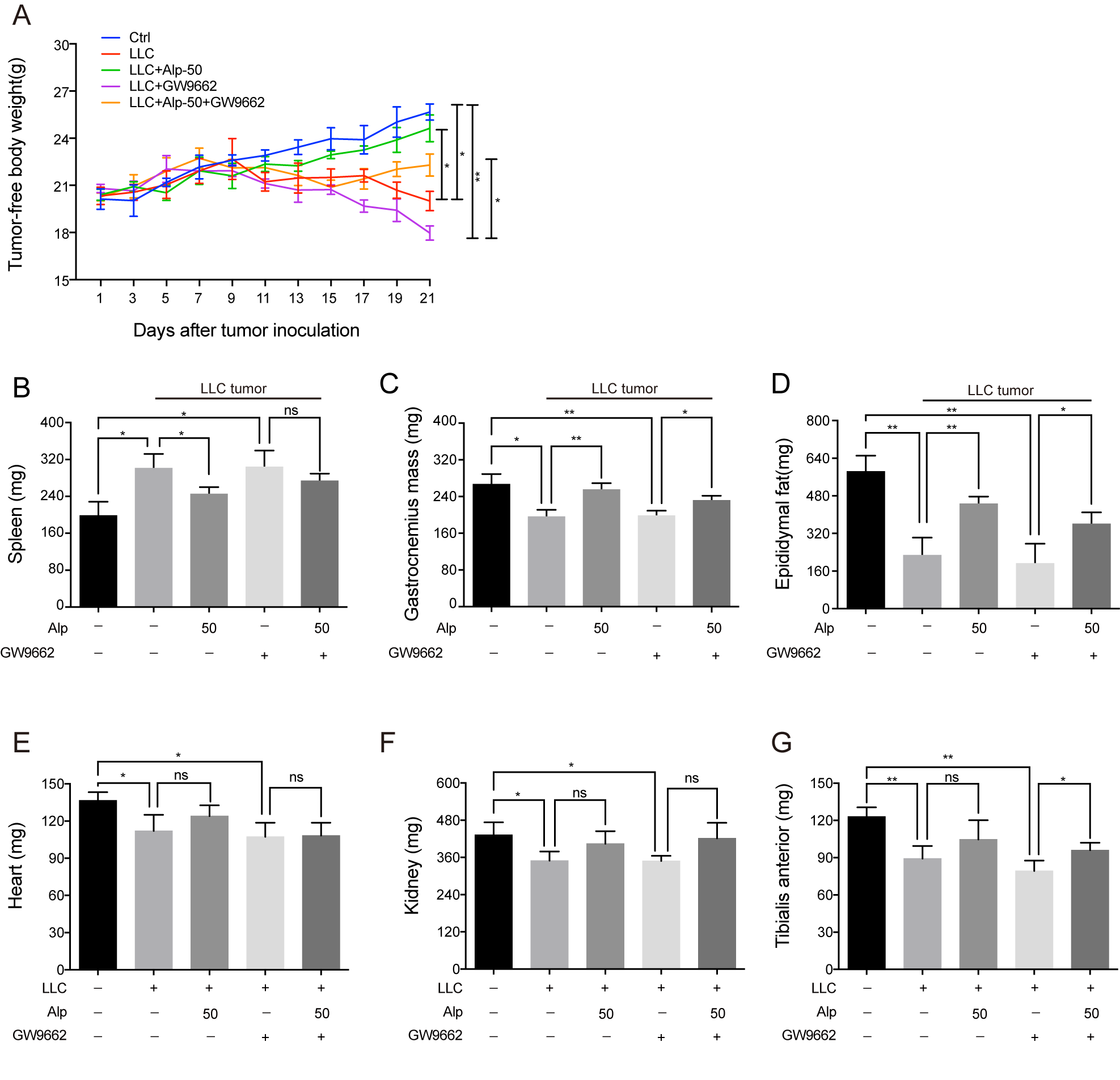 |
| --- |

**Figure S2** Alp activates PPARγ in muscles of LLC tumor-bearing mice. Effects of PPARγ on (A) tumor free body weight during the 21-day study were explored. (B-D) spleen, gastrocnemius and epididymal fat mass, and (E-G) heart, kidney and tibialis anterior muscle mass revealed are raw values. *P < 0.05, **P < 0.01, ***P < 0.001, ****P < 0.0001 versus control. ns: not significant. n = 10 mice/group.


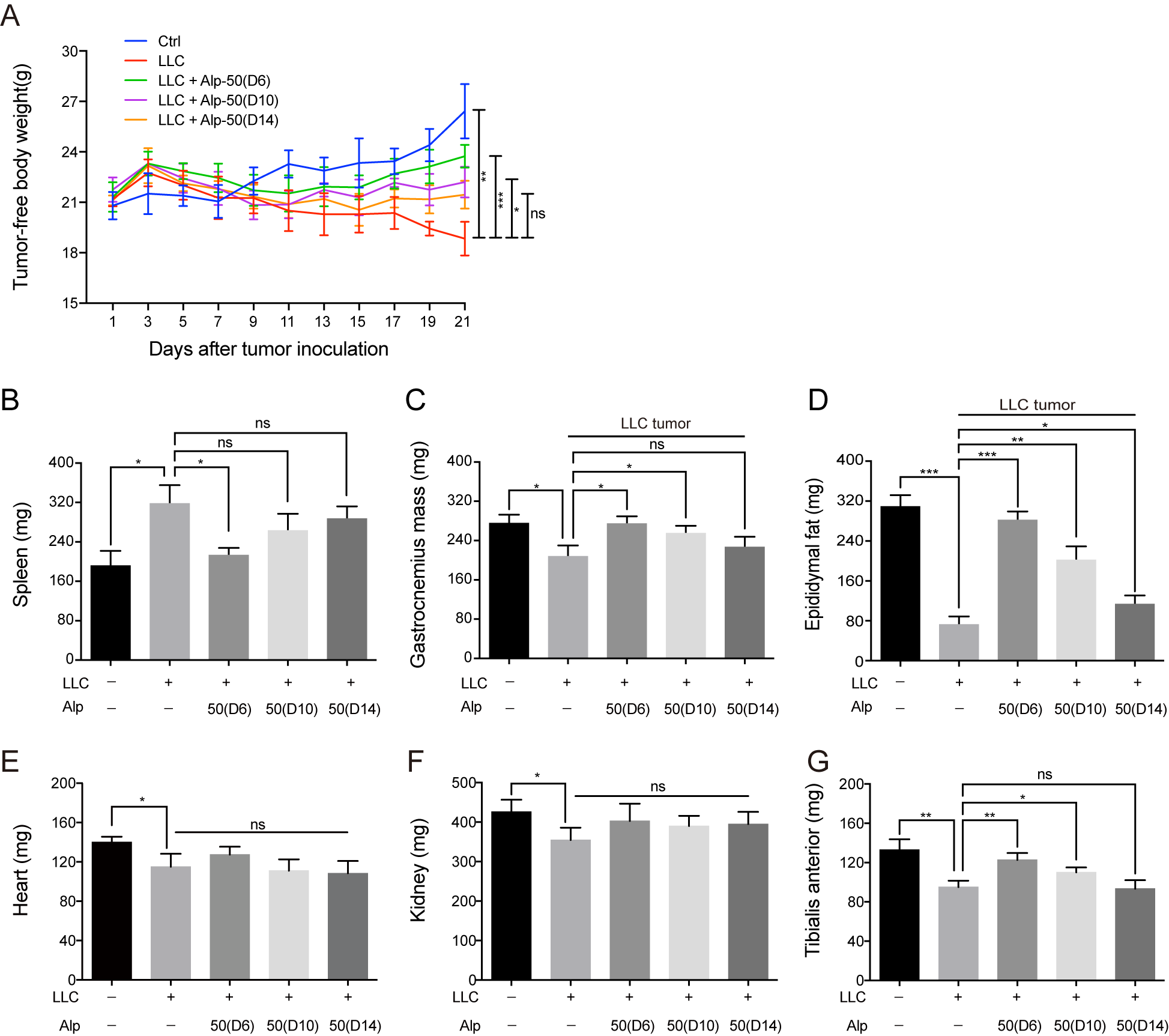


|  |
| --- |

**Figure S3** Effects of delayed treatment with Alp until late stages of tumor and cachexia process in LLC tumor–bearing mice. Effects of delayed treatment with Alp on (A) tumor-free body weight during the process of the 21-day study were analyzed. (B-D) spleen, gastrocnemius and epididymal fat mass, and (E-G) heart, kidney and tibialis anterior muscle mass showed are raw values. *P < 0.05, **P < 0.01, ***P < 0.001, ****P < 0.0001 versus control. ns: not significant. n = 10 mice/group.
